# Wnt/β-catenin Pathway-Mediated PPARδ Expression during Embryonic Development Differentiation and Disease

**DOI:** 10.3390/ijms22041854

**Published:** 2021-02-12

**Authors:** Tabinda Sidrat, Zia-Ur Rehman, Myeong-Don Joo, Kyeong-Lim Lee, Il-Keun Kong

**Affiliations:** 1 Department of Animal Science, Division of Applied Life Science (BK21 Four), Gyeongsang National University, Jinju 52828, Korea; tabindasidrat06@gmail.com (T.S.); jmd1441@gmail.com (M.-D.J.); 2 Department of Microbiology, Hazara University, Mansehra 21310, Pakistan; Zia7487@gmail.com; 3 The King Kong Corp. Ltd., Gyeongsang National University, Jinju 52828, Korea; 0920-0728@hanmail.net; 4 Institute of Agriculture and Life Science, Gyeongsang National University, Jinju 52828, Korea

**Keywords:** Wnt/β-catenin signaling, PPARδ, embryonic development

## Abstract

The Wnt/β-catenin signaling pathway plays a crucial role in early embryonic development. Wnt/β-catenin signaling is a major regulator of cell proliferation and keeps embryonic stem cells (ESCs) in the pluripotent state. Dysregulation of Wnt signaling in the early developmental stages causes several hereditary diseases that lead to embryonic abnormalities. Several other signaling molecules are directly or indirectly activated in response to Wnt/β-catenin stimulation. The crosstalk of these signaling factors either synergizes or opposes the transcriptional activation of β-catenin/Tcf4-mediated target gene expression. Recently, the crosstalk between the peroxisome proliferator-activated receptor delta (PPARδ), which belongs to the steroid superfamily, and Wnt/β-catenin signaling has been reported to take place during several aspects of embryonic development. However, numerous questions need to be answered regarding the function and regulation of PPARδ in coordination with the Wnt/β-catenin pathway. Here, we have summarized the functional activation of the PPARδ in co-ordination with the Wnt/β-catenin pathway during the regulation of several aspects of embryonic development, stem cell regulation and maintenance, as well as during the progression of several metabolic disorders.

## 1. Introduction

The mammalian pre-implantation period is one of the critical and unique phases during early embryonic developmental processes. A transition from single-cell zygote to the blastocysts stage involves a series of crucial events that lead to the initiation of cell lineage specification and differentiation into the inner cell mass (ICM) and trophectoderm (TE) [1,2]. These intricate developmental processes are regulated by the activation of several intracellular signaling cascades. Among these pathways, Wnt signaling is an evolutionarily conserved pathway and has been well-known to play an essential role during all stages of vertebrate embryogenesis. Wnt signaling has been primarily divided into two types, namely canonical and non-canonical pathways. The involvement of Wnt signaling is well-characterized in multiple cellular processes, such as cellular proliferation, differentiation, the establishment of cell polarity, regulation of stem cell renewal during embryogenesis, and tissue homeostasis [3,4]. Wnt participates in all of these intricate processes either directly or by a crosstalk with other signaling pathways. The peroxisome proliferative activated receptor delta (PPARδ) of the nuclear superfamily receptor, which primarily regulates the lipid metabolism, also plays a crucial role during embryogenesis in conjunction with Wnt/β-catenin signaling [5,6]. The dysregulation of these signaling pathways causes congenital malformations such as cancer, osteoporosis, and different metabolic disorders [4,7]. The focus of this review is Wnt/β-catenin mediated PPAR delta signaling during the developmental processes.

**Figure 1:**
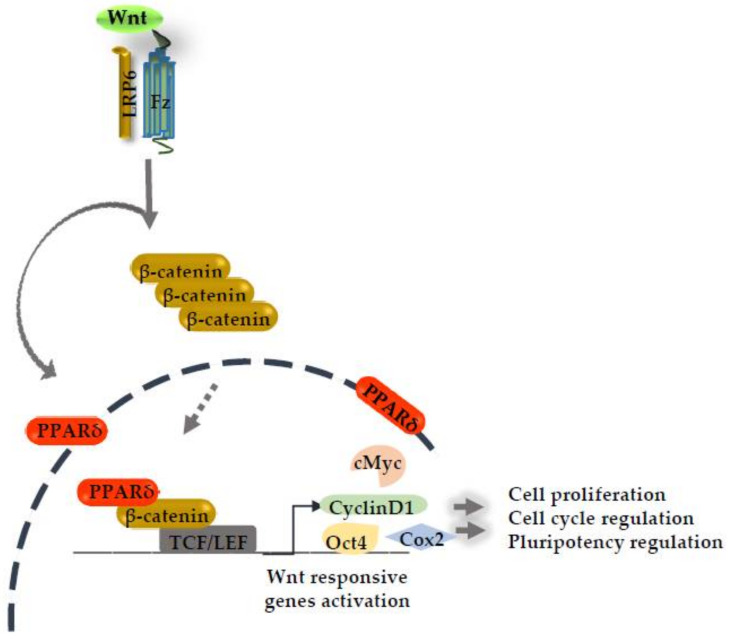
Canonical Wnt signaling activation induces the peroxisome proliferative activated receptor delta (PPARδ) expression. PPARδ synergizes with the β-catenin transcriptional activation complex in the nucleus and boosts the expression of several Wnt responsive genes.

The canonical Wnt pathway is called a β-catenin dependent pathway. It depends upon the subsequent accumulation of the activated β-catenin level in the cytoplasm. In the presence of the canonical Wnt ligand, a multi-protein complex (Wnt/Fzd/Lrp6) is formed at the membrane that triggers the downstream signaling. This complex directs the β-catenin destruction complex (Gsk3/APC/Axin) to the membrane and leads to the stabilization of β-catenin levels in the cytosol [3,8]. The accumulated β-catenin is translocated to the nucleus, where it makes a transcriptional co-activator complex with DNA binding protein T cell factor/lymphoid enhancer factor (TCF/LEF) and modulates the activity of several Wnt target genes [3,9]. The expressions of these genes are cell type and lineage dependent, and include genes responsible for the regulation of developmental processes, cell cycle regulation, and cell proliferation [8,10]. The emerging role of PPARδ has been reported during the pre-implantation period of embryonic development, cancer, and several metabolic disorders in response to β-catenin/TCF4 signaling [5,11]. PPAR family members, such as PPARα, PPARγ, and PPARδ, are activated by a multitude of agents. In these three PPARs, PPARγ and PPARδ are particularly activated in response to prostaglandins (PGs) [12]. The production and feedback of PGs are dependent on the activity of the Cycloxygenase enzyme (Cox1 and Cox2), which contains the response element-binding region for PPARs [12,13]. Importantly, the Cox2 promoter binding site was revealed to be present on the TCF/LEF transcription binding protein. Therefore, the activation of the Wnt/β-catenin pathway directly influences the expression of Cox2 to impact several aspects of development and reproduction [14]. These observations led us to highlight the currently known association of PPARδ either directly or indirectly with the Wnt/β-catenin pathway for the regulation of several important processes during development and differentiation, as illustrated in (Figure 1).

## 2. Impact of Wnt/β-Catenin Signaling and PPARδ Activation on Cell Lineage Specification and Pluripotency during Early Embryonic Development

A spatially defined and well-controlled Wnt signaling pathway orchestrates normal embryonic development. This process is initiated at fertilization and is continued until the formation of a complete organism [1,15]. To reach the two committed cell lineages, embryonic development undergoes two successive differentiations [16,17]. The multiplication of these cells leads to the first differentiation at the morula stage and consequently establishes the ICM and TE layer. The second wave of cell differentiation distinguishes the ICM into a pluripotent epiblast (Epi) and an extraembryonic tissue primitive endoderm (PrE) [2]. Further expansion and outgrowths of the ICM to support the in vitro maintenance of the embryonic stem cell (ESC) culture depend on the expression of core pluripotency factors [10,18]. The Wnt/β-catenin pathway plays a pivotal role in maintaining the pluripotency both in vivo and in vitro in human and mouse ESC cultures. The pluripotency of ESCs is maintained by the expression of Yamanaka factors such as Oct4, Nanog, Sox2, and Klf4 [10,19,20,21]. The β-catenin interacts with Oct4 and c-Myc and enhances their expression in a Tcf-dependent manner [18,22]. Several in vivo and in vitro studies showed that the presence of Oct4 and c-Myc expression is crucial during the pre-implantation period of embryonic development, maintenance of cell proliferation, and ICM identity [5,23]. Recently, a study on mouse and bovine embryonic development reported that increased Wnt/β-catenin signaling is also manifested by the up-regulated expression of the PPARδ gene in both the ICM and TE cell population [5,6]. The PPARδ expression maintains the higher cell index ratio by sustaining the proliferative signaling in response to β-catenin/Tcf4 signaling [11]. In vitro studies of mouse and bovine embryos showed that Wnt3 stimulation simultaneously increases the PPARδ and c-Myc expression in ICM and trophoblast stem cells (TSCs) and supports the progressive proliferation event during early development [5,6]. These pieces of evidence highlight the functional importance of PPARδ expression during embryonic development and the maintenance of ESCs’ pluripotent state in coordination with the Wnt/β-catenin pathway.

The early developmental processes are governed by the regulated processes of cellular proliferation, pluripotency maintenance, cell migration, and differentiation [1,7,10]. In vitro ESC studies demonstrated the essential requirement of Wnt signaling to regulate all of these processes. Constitutive expression of Wnt signaling with the help of β-catenin and Gsk3i (glycogen synthase kinase inhibitor; CHIR99021) efficiently restores the undifferentiated state of ESCs [18,19]. In our recent study, we observed that enhanced propagation of Wnt signaling with the addition of 6-Bio (Gsk3i) increases the ICM cell proliferation index and leads to better quality and yield of bovine blastocysts [6]. The cellular proliferation and the quality of the developing embryos were aberrantly affected upon the addition of the PPARδ specific inhibitor GSK3787. Interestingly, the Wnt over-activated condition reversed this effect and rescued the PPARδ expression [6]. PPARδ is constitutively expressed in the nucleus. Activation of Wnt brings PPARδ and β-catenin into proximity, where they potentially make a complex with activated β-catenin upon Wnt stimulation and regulate embryonic development [6]. However, direct physical interaction of PPARδ with β-catenin via chromatin loop formation has also been reported during vascular endothelial growth factor (VEGF) gene transcription in colon cancer [24]. The formation of a transcriptional activator complex between Tcf/Lef/β-catenin/PPARδ promotes the Wnt target gene’s expression [24]. Interestingly, direct Wnt target genes, such as CyclinD1 and c-Myc expression, which are influenced by the β-catenin/Tcf transcriptional activation complex, are also enhanced synergistically upon PPARδ activation [6]. These observations suggest that PPARδ has a strong functional association with Wnt/β-catenin signaling. Furthermore, PPARδ strongly couples with c-Myc expression in the ICM as well as in TS cells during the elevated Wnt condition [5,6]. These findings define the correlation between Wnt/β-catenin signaling and PPARδ activation. The connection of Wnt/β-catenin signaling and PPARδ regulates the cell proliferation ability and pluripotent potential of ICM in the early stages of embryonic development. 

## 3. The Role of Wnt/β-Catenin Signaling and PPARδ Activation in Implantation Potential and Invasion

The role of Wnt signaling in governing pre-implantation development is somewhat controversial. However, β-catenin was detected from the 2-cell stage to the blastocyst stage of mouse embryos, suggesting that Wnt/β-catenin signaling has a functional role in driving the early processes of embryogenesis [15]. The development of an embryo until the implantation of a competent blastocyst is key for the successful outcome of the pregnancy. The participation of Wnt signaling and PPARδ activation in the promotion of blastocyst implantation potential has been demonstrated in several species during in vitro embryonic development [25,26]. Recently, it was documented that in vitro-produced bovine blastocyst implantation potential is enhanced via Wnt/β-catenin-mediated PPARδ activation, which explains the synergistic actions of PPARδ with Wnt signaling [6]. In the TS cell culture model for implantation, the presence of activated β-catenin in the TE cells is responsible for the promotion of the migratory abilities of the TE cells via the up-regulation of c-Myc and PPARδ expression [5]. It is important to highlight that β-catenin also functions as a cell–cell adhesion protein that regulates cell migration during embryonic development and metastasis in carcinomas [27,28]. Therefore, the high level of β-catenin accumulation in the TE cells increases the cell migration and invasion capacity of the cells [5]. Cell migration and differentiation are essential processes during early embryogenesis [28]. Moreover, the elevated expression of PPARδ increases the cell invasiveness and progression of metastasis [11]. PPARδ expression is strongly co-localized with β-catenin in the early phases of development [6], suggesting that it may influence the adhesive and migratory properties of the TE cells associated with β-catenin. The mechanism through which the up-regulated PPARδ expression in response to the activated Wnt condition regulates the adhesive and migratory properties of epithelial TE cells needs further investigation. It will be interesting to see whether elevated PPARδ expression can modulate the functions of the β-catenin adhesions junctional protein complex so as to reveal its wider impact on development and differentiation. 

## 4. Wnt Signaling and PPARδ Role in Trophoblast Differentiation and Placentation

The development of placenta and maintenance of its integrity is another crucial aspect of normal embryonic development and successful pregnancy outcome. Numerous reports highlighted the critical involvement of the Wnt/β-catenin signaling pathway as well as the significance of PPARδ functions during trophoblast differentiation and placentation [29,30,31]. The Wnt and PPARs family performs several coordinated functions to regulate various developmental processes, but the understanding of the link between Wnt and PPARδ-mediated regulatory processes has been lagging behind. The focus of this review is to furnish specific information and provide potential hints about the synergistic role of Wnt/β-catenin and PPARδ signaling in orchestrating the essential features of embryonic development. 

Placenta originate from the outermost trophectoderm layer of a blastocyst that differentiated into several trophoblast cell types [31,32]. Differentiation and proliferation of the trophoblast during the initial stages of blastocyst implantation and invasion yielded a multitude of primary and secondary giant cells. These trophoblast giant cells participate in a number of regulatory and secretory processes crucial for the development of both fetal and maternal placental compartments as well as the remodeling of maternal uterine stromal lining [32]. During these sequential steps of placental development, the presence of Wnt signaling is critically important. Several mice knockout studies provided evidence that Wnt/β-catenin signaling is indispensable for early placentation [33]. The development of embryonic stem cells in response to Wnt3a leads to the formation and differentiation of trophoblast giant cells by the induction of lymphoid enhancer factor-1 (LEF-1)-dependent caudal type homeobox transcription factor 2 (CDX2) expression [34]. PPARδ, which is a β-catenin target gene, is also an important mediator of trophoblast differentiation and placentation [35]. Notably, the activation of PPARδ accelerates the giant cell differentiation in vitro. Furthermore, the homozygous deletion of PPARδ^-^/^-^ in mice inhibited trophoblast differentiation towards the giant cells that substantially impact placental development [35]. Deficiency of the PPARδ gene caused severe placental defects by hindering the placental tissue from undergoing proper morphogenesis and resulted in frequent embryonic lethality at stages E9.5 to E10.5 in mice [36]. Placental development involves a complex tissue remodeling process, supported by branching morphogenesis, chorio-allantoic fusion, labyrinth development, and placental angiogenesis [32]. Genetic loss-of-function studies of Wnt pathway components and PPARδ showed that striking similarities exist in the placentation development defects. For example, deletion of the Wnt transcription factor both for TCF-1 and LEF-1 in mice caused a defect in labyrinth formation due to a failure of chorionic-allantoic fusion [33,34]. Knock-out of the Wnt receptor FZD5 caused mortality of mice embryos at E10 due to less vascularized placentae [33]. Likewise, the homozygous null mutant of PPARδ embryos failed to survive beyond E10 due to defects in the labyrinthine trophoblast compaction and reduced vascularization of placentae [31,36]. PPARδ induces the expression of vascular endothelial growth factor A (VEGFA), a key regulator of vasculogenesis, and promotes vascular permeability. Importantly, PPARδ activates the transcription of VEGFA via β-catenin-mediated chromatin regulation [24]. PPARδ induces the vascularization function during placenta development and might rely on β-catenin-mediated gene transcription regulation; alternatively, this effect may be cell-context-specific and needs further elucidation. This evidence indicates that Wnt/β-catenin signaling and PPARδ have been critically implicated during trophoblast differentiation and development of placental integrity. PPARδ, which in response to Wnt-activated conditions performs several synergistic functions in coordination with β-catenin to regulate proliferation and differentiation events during embryonic development [5,6,35], likely provokes the notion that the Wnt/β-catenin pathway might exert some of its effect via PPARδ activation during placentation. In order to suggest a possible mechanism and to delineate a specific interaction of PPARδ expression with the Wnt/β-catenin pathway, further studies are required to explore the requirement of PPARδ activation in response to Wnt stimulation during placentation. 

## 5. Wnt/β-Catenin and PPARδ Signaling Regulate Cell Proliferation Events during Embryonic Stem Cell Maintenance 

Stem cells are characterized by self-renewal by maintaining the pluripotency and the proliferation potential of the progenitor cells [37]. This phenomenon is essential during normal tissue homeostasis and developmental processes [8]. The β-catenin-dependent Wnt signaling pathway is one of the major regulators of ESC maintenance and adult mammalian tissue homeostasis [18,38]. In addition to the Wnt/β-catenin pathway, another important factor for the regulation of the self-renewal characteristic is the leukemia inhibitory factor (LIF). Several studies reported crosstalk between LIF and Wnt/β-catenin signaling [10]. A downstream target of LIF signaling is the signal transducer and activator transcription 3 (STAT3) protein, which is activated by the stimulation of Wnt signaling [18,37]. It was also demonstrated that in mouse ES cell cultures in the absence of β-catenin the high expression of LIF can retain the pluripotent characteristics of the stem cells. Conversely, the addition of the GSK3 inhibitor, which stabilizes the β-catenin level, reduces the requirement of LIF for ES cell pluripotency and self-renewal [39,40]. Interestingly, in some contexts the activated expression of PPARδ was reported to increase the phosphorylation of STAT3, an important factor of the LIF signaling pathway during mesenchymal stem cell culture [41]. As in LIF signaling, cell cycle regulators are also key factors for maintaining the stemness of the cells [37,42]. Loss of the self-renewal potential of the proliferating stem cells is directly proportional to the reduced expression of the components of cell cycle machinery [10]. Studies have reported that cell cycle regulators and pluripotent activities of stem cells are regulated by the same set of genes and pathways [42]. The Wnt/β-catenin pathway is also an important factor for the control of cell cycle regulation [10]. Activated Wnt/β-catenin signaling increases the expression of CyclinD1, c-Myc, and PPARδ during embryonic development as well as during the progression of cancer [5,6,43]. Importantly, the selective inhibition of PPARδ function by GSK3787 reduces CyclinD1 and c-Myc expression during in vitro embryonic development [6]. These observations reveal the involvement of PPARδ in cell cycle progression and the pluripotent potential of stem cells. However, the molecular evidence underlying the absolute requirement and direct relationship of the Wnt/β-catenin pathway and PPARδ expression during ES cell pluripotency maintenance is still exotic. Further study is needed to elucidate the link between PPARδ and other signaling pathways. 

## 6. Wnt/β-Catenin Signaling and PPARδ Implication during Metabolic Disorder and Cancer Progression

Wnt/β-catenin signaling represents a major pathway upon which various signals converge to influence metabolism and cancer progression [44]. Imbalance or excess of lipids in skeletal muscles or adipose tissue leads to the development of metabolic syndrome [44,45]. The PPARs family is known as a central regulator of lipid metabolism and energy homeostasis. Wnt and PPARδ signaling has been implicated as playing an important role in the normalization of fat accumulation, the reduction of adiposity and insulin sensitization [36,44,45]. Recently, molecular cross-talk between PPARδ and Wnt signaling was documented, which potentiates osteoblast differentiation by suppressing pre-adipocyte differentiation [46]. A few pieces of compelling evidence indicated PPARδ as a Wnt target gene [5,6,47,48]. Regarding the regulation of tissue metabolic reprogramming, PPARδ also serves as a potent candidate of the Wnt/β-catenin pathway and plays a role in the modulation of fatty acid metabolism and adipocyte production [46]. Wnt signaling activity induces PPARδ expression, which directly interacts with β-catenin/TCF/LEF transcription factors and enhances the expression of lipoprotein lipases, such as fatty acid translocase (FAT), fatty acid binding protein (FABP), and carnitine palmitoyl-transferase 1 (CPT1) [46,49]. The synergistic action between Wnt/β-catenin signaling and PPARδ has been more strongly supported when the treatment of mesenchymal stem cells with the specific PPARδ agonist GW501516 resulted in the amplification of Wnt ligand-induced nuclear β-catenin accumulation and in Wnt co-receptor LRP5 expression. Moreover, chromatin immunoprecipitation further revealed a direct binding of PPARδ on the LRP5 promoter in MC3T3 cells [46]. These findings suggest a positive feedback loop between both pathways during the regulation of several developmental and metabolic functions. 

So far, PPARδ has been majorly implicated in lipid catabolism in adipose and muscle tissue by enhancing fatty acid oxidation (FAO). This molecular cross-talk between Wnt signaling and PPARδ has been implicated in many metabolic syndromes, such as obesity, diabetes and the development of cancer [36,46,50]. The Wnt/β-catenin pathway is known as an oncogenic signaling cascade, and it promotes the development and progression of tumors by affecting the tumor cell metabolism [51]. PPARδ also emerges as a Wnt downstream target gene in the progression of several types of cancer. Elevated PPARδ expression has been observed during the progression of colorectal cancer in response to aberrant β-catenin activation, which is caused by adenomatuous polyposis coli (APC) mutation [11,36]. Elevated PPARδ expression promotes fat oxidation near adipose tissue surrounding the tumor, thus providing a sufficient supply of energy substrates for the development and proliferation of the tumor microenvironment [11,46]. However, the function of PPARδ in conjunction with β-catenin during the development of colon carcinogenesis is still uncertain [11,52]. To reveal a consistent understanding, PPARδ expression and function needs to be more critically examined in normal and cancerous tissue in order to identify its critical nodes in Wnt signaling for the treatment of various metabolic disorders and the inhibition of tumorigenesis.

## 7. Future Perspectives 

**Figure 2:**
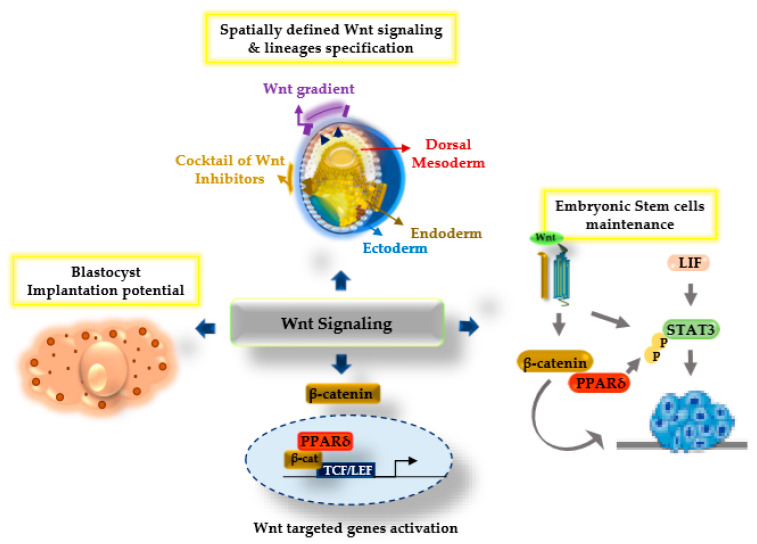
Model diagram illustrating the Wnt/β-catenin signaling and PPARδ synergistic functions regulating several aspects of development. Upon Wnt stimulation, the β-catenin and PPARδ transcriptional complex with the T cell factor/lymphoid enhancer factor (TCF/LEF) plays a crucial role during the specification of embryonic lineages, and enhances blastocyst implantation potential and the regulation of embryonic stem cell maintenance.

Wnt signaling has a broad range of roles in the control of early embryonic developmental processes. Wnt/β-catenin signaling is remarkably known for the regulation of cell proliferation and differentiation events during early embryogenesis and stem cell maintenance. This multitude effect of Wnt signaling may involve crosstalk with other signaling components. The above model diagram (Figure 2) highlights the synchronized actions of PPARδ with β-catenin upon Wnt stimulation and suggests that these actions are an important factor in supporting Wnt-regulated functions during development and differentiation. However, to see the Wnt signaling-dependent cellular outcome, a detailed analysis of PPARδ interaction with β-catenin as well as with TCF/LEF transcription factors is required. Further studies using the knockdown and knockout of the PPARδ gene are needed to better understand the mechanistic action of PPARδ in coordination with Wnt/β-catenin signaling in different aspects of development and differentiation. A better understanding of Wnt/β-catenin signaling in coordination with PPARδ activation will have a broader impact on biology and medicine.

## Abbreviations

APC	Adenomatuous Polyposis Coli
CDX2	Caudal Type Homeobox Transcription Factor 2
CPT1	Carnitine Palmitoyl Transferase-1
Epi	Epiblast
ESC	Embryonic Stem Cells
FABP	Fatty Acid Binding Protein
FAO	Fatty Acid Oxidation
FAT	Fatty Acid Translocase
FZD5	Frizzled (class receptor 5)
GSK3i	Glycogen Synthase Kinase 3 Inhibitor
ICM	Inner Cell Mass
KLF4	Krupple Like Factor 4
LEF-1	Lymphoid Enhancer Factor-1
LRP5	Low density Lipoprotein Receptor related Protein-5
LIF	Leukemia Inhibitory Factor
MC3T3	Murine Calvarial Osteoblast Cell Line
OCT4	Octamer Binding Transcription Factor 4
PPARδ	Peroxisome Proliferative Activated Receptor Delta
PrE	Primitive Endoderm
SOX2	SRY-Box Transcription Factor 2
STAT3	Signal Transducer Activation Factor 3
TE	Trophectoderm
TCF	T Cell Factor
VEGF	Vascular Endothelial Growth Factor
